# American Medical Association, Section on Stomatology

**Published:** 1904-03

**Authors:** 


### Reports of Society Meetings.

### AMERICAN MEDICAL ASSOCIATION, SECTION ON STOMATOLOGY.

(Continued from Vol. XXIV., page 704.)

### DISCUSSION OF DR. CHITTENDEN'S PAPER, "IS THE REALIZATION OF REASONABLE IDEALS IN DENTAL EDUCATION NEAR AT HAND?"

(For Dr. Chittenden's paper, see Vol. XXIV., page 524.)

*Dr Eugene S. Talbot.*—There is perhaps no one in the country so well posted on the management of dental colleges as the essayist. He is one of the foremost men who has been looking forward to the future of dentistry, and has done a great deal in the way of legislation. He would have been here at this meeting but for the fact that the Legislature is now in session, and changes are being brought about in the State law in Wisconsin. He wished me to extend his thanks to the Association for its courtesy in having his paper read, and regrets that he could not be here.

There is no doubt but that there is a good deal of underhand work going on in some of our dental colleges. I have in mind a city where there are three or four dental schools from some of which agents are sent out to solicit students, and to whom a percentage of the fees are paid for procuring them. There are many other glaring deficiencies in the methods of obtaining students. I was a little surprised that more was not said along these lines.

I understand that it is the intention of the National Board to bring this subject before their meeting for the purpose of discussing some of these points. I believe that the time has arrived when the Departments of Dentistry connected with universities will have a large influence. Now that the universities have a method for the purpose of uniforming the education in all their departments, and also for the purpose of raising the standard of qualification for admission to these different departments, I believe the time is near at hand when the graduate of dentistry in this country will be on an equal standing with the graduates of other specialties.

*Dr. M. H. Fletcher,* Cincinnati.—I do not know that I have much to say, except that I am interested in dental education and the higher standard that some of us conceive of being brought about. The author of the paper is more familiar with the progress in dental education than I, but as a member of the State Board of Dental Examiners for five years, it is a matter of great chagrin to me to know that our laws compel the examiners to give such elementary examinations that what I would consider a first course student in a college when I was teaching either dentistry or medicine could pass an examination. There is every reason from my stand-point for extreme effort in raising the standard, and no doubt such men as the essayist and those interested in bringing it about need the co-operation of all good men in this work. I sincerely trust that the day is not far distant when the standard of efficiency may be raised.

*Dr. M. L. Rhein,* New York.—No one more appreciates the good work that has been done in forwarding dental education in this country than I do that of Dr. Chittenden. It goes without saying how much we are in sympathy with the elevation of the standard of qualifications for admission to a dental educational school. I feel personally that Dr. Chittenden made his paper a little weaker than he intended when he spoke of the smaller colleges being irretrievably hurt by the length of the course, because I feel very much on this subject as was expressed by Dr. Billings, the president of the Association, in his opening address, that the time has come when the smaller educational institutions must go out of existence. There is no use for them at the present time. I would even say that I think the idea of proprietary educational institutions is a mistake. I think this Section should come out flat-footed on the question of men enjoying a salaried position due entirely to the income of the institution with which they are connected. That has invariably been one of the worst features connected with proprietary dental education in this country. If the efforts to raise the standard of dental education is a blow at this sort of institution, it is a blow that is well-merited and in which personally I am in full sympathy, because the sooner they are eradicated and destroyed, the better for the interests of the country at large. How much better it would be if there were fewer institutions in which there was a distinctive salaried position of an amount to enable the proper teacher to be selected, irrespective of the necessity of earning their living by the practice of their profession. It is impossible at the present day for the real professor of any branch in a dental institution to be engaged in the active practice of his calling. His attention to practical dentistry should be confined to the infirmary division of the institution with which he is connected. This is the idea we have to look forward to in dental education. It makes little difference, to my mind, how much we raise the standard, if we fail to accomplish the real results.

There is just one little point of difference that I have with Dr. Chittenden in his paper, that is, the words of criticism that he makes at institutions of character for keeping themselves advertised, as he says, in the eyes of the public, advertising their superiority over the smaller institutions. I hardly believe, if I could talk this matter over with Dr. Chittenden, that he would see this in the light in which he appears to, judging from his paper. To my mind, it is one of the forms that is necessary to use to drive out of existence the inferior institution. It is impracticable in my view to compare any one of our great institutions with an advertising dental parlor; so I think Dr. Chittenden has made rather an unfortunate mistake in a comparison of this kind. These large institutions are constantly before the eyes of the public in the public print. A very necessary form of the education of the young men to-day is the athletic surroundings of the university. There are differences of opinion regarding their value, but at the present time they have the sympathy and the accord of the American people. I myself believe that they play an important part in the welfare of our educational institutions. I believe they do good, and can do harm. Devotion of time to them to the neglect of studies is the same evil which besets every young man in all surroundings of life. A great deal of the advertising of departments of universities is of such a nature as is not directly sought for by these universities. It is worked up by the individual parties, and I personally see no harm in it. I believe this is a sort of competition which will bring out better features from the opposite institutions. I must say that I differ from my friend Dr. Chittenden on that one point.

*Mr. T. Constant,* Scarborough, York, England.—I have so little knowledge of the condition of education in this country that I am hardly in a position to speak upon the subject. At the same time, speaking generally on the question of education, a subject to which I have given some attention, I think the most necessary thing in considering the educational conditions of any kind is to insist upon a very high standing of preliminary education. The time that is necessary to give to the application of technical knowledge, when the mind is very receptive, as in the case of medicine and surgery, and especially in dentistry, is so largely devoted to the requirements of special knowledge that one's general knowledge is liable to be neglected. I think, therefore, that prior to examinations of special studies one's general education should be as complete as possible. I feel that the fewer educational centres we can have, the better. The facilities for travel now are such that distances are not the consideration they used to be, so that one great objection to having centres of education is removed. I therefore think that great harm is done, and I believe, from my little knowledge, particularly so in this country, by multiplication of centres of education. On the same lines I believe, myself, in the uniform standard of examinations. I think if all the States of America united and had one national standard in examinations to which it became necessary for every student to present himself before being licensed to practise it would be a good thing. He could then succeed in taking the ordinary university degree. License to practise, I believe, should be determined by national examining centres.

*Dr. W. E. Walker,* New Orleans.—I believe the idea of the formation of great universities with dental departments is the correct one, and that the working out of the plan as it is being done in Harvard is really what it ought to come to; that there should be the unification of preliminary requirements for all the different departments instead of having one for medicine and another for dentistry, the basal studies being taken alike by all, and capped by the special work according to the field a man intends to make his life-work.

The question of preliminary requirements is a very difficult one to handle. Because it was found that when the dean examined the students he was so influenced not only by his desire for the college to grow, but by the forces brought to bear upon him by other teachers, that he would sometimes admit students that ought not to be admitted, it was thought that that would be overcome by the requirement that the dean should no longer examine students for entrance, but that the county superintendent of education should have the matter in hand. The law does not specify whether it shall be the superintendent of the county in which the school is located or in which the student resides. The result is that there is a different standard. We find many boys coming from the country where the standards are low and where the county superintendent will allow himself to be influenced by personal feeling and give him a certificate which he would not have received had he come to the educational centre to be examined by the county superintendent in which the school is located where the standard would be higher. I have an instance in mind in which a student was examined and refused by the examiner of the county in which the school was located, and went home and was passed by the superintendent of the county in which the student resided. There must, therefore, be some change in that direction. Just how it is to be done I do not know. Regarding the universities, if there could be a national body to examine all applicants it would seem to work more satisfactorily than any other means.

A paper entitled "The Vasomotor System of the Pulps," with exhibition of slides, was read by Eugene S. Talbot, M.D., D.D.S., of Chicago.

(For Dr. Talbot's paper, see page 89.)

DISCUSSION.

*Dr. M. H. Fletcher*, Cincinnati.—There is not much which occurs to me in the way of discussion of this paper except to commend what has been said. The subject of the nerve distribution of the pulp seems to be greatly neglected, and it is a matter of great pleasure to know that it has been taken up by such physicians as Drs. Talbot and Latham, because through these studies one may be able to find out why its tendency is to recede from any attempt at investigation. I do not think the doctor has given any definite theory, except to show us the distribution of the nerves about the vasomotor system. If I have overlooked that point I shall be glad to have the doctor tell me whether his theory shows anything aside from discovering to us the anatomy as it now is. I am certainly very much pleased to have this subject taken up, because it will result to our knowledge.

*Mr. Constant*, England.—I would like to express my appreciation of Dr. Talbot's very valuable paper. I regret that he did not read the whole of it, because it would be of great interest to me to have learned the later views of American writers on the subject of the vasomotor system. The photographs and micrographs were extremely interesting, and the feature that struck me more particularly was the great continuity of the non-medullated fibres that the photographs showed. It is of particular interest to me, because the work that I have recently been doing in connection with the pulp comes into touch with that work in one or more ways. From clinical reasons I have for some years been under the impression that the so-called odontoblasts have more to do with the vasomotor system than the mesoblasts. They have, in my opinion, very little to do with the formation of dentine, excepting so far as regulating the blood-pressure. They undoubtedly assist, but not in the direct manner in which most English dental anatomists have described.

The paper, therefore, of Dr. Talbot has been of great interest to me, and to have heard that alone I should consider my visit here fully repaid.

*Dr. Gilmer.*—The subject is not one that I can discuss. I think, however, that we are greatly indebted to Dr. Talbot for this work and the success he has met with. The facts are before us. He shows us what is in the pulp, and I think it is not a matter which we can criticise.

*Dr. Eames.*—I am much interested in this paper, because I can see how one may take advantage of the idea therein expressed clinically. For instance, there is no place in the body where we can see the control of the nervous system over the tissues as in the nose. I believe that in many inflammatory conditions of the pulp we may control these conditions, as we understand what could influence the general nervous system. I have had a patient say to me, "When I am tired I have a pain in my tooth." I hardly realized at the time what that meant, but I can see now how exhaustion of the nervous system will so influence the control of the blood-supply in that organ that it will be paralyzed in a way so that it will let blood in there and cause pain. I can see also how, in a subacute inflammatory condition, a person whose nervous system has been exhausted may acquire a chronic state of inflammation in the pulp by paralyzing these fibres so that the blood-vessels themselves become permanently stretched. Therefore, I do think that these demonstrations have a clinical value in this respect.

*Dr. H. E. Belden.*—I feel a good deal like the student who wrote to the physician, asking how he would tell when he had reached pulp. The physician wrote back that the patient would tell him. I have never made any minute study of the anatomy of the pulp, but I can see the good that would arise from it, and I am very glad to have had the pleasure of seeing the paper demonstrated.

*Dr. Jules J. Sarrazin,* New Orleans.—I have nothing to add to the discussion. I thoroughly appreciate the work done, the magnitude of which is appalling. I agree with the expression that the amount of knowledge given may become of great usefulness in a better understanding of the physiological conditions. The paper presented by Dr. Talbot reveals an enormous amount of thorough research, but in my opinion, we are not yet in position to fully profit by the work. We have to get a greater elucidation of the work and the deductions which may be drawn from that work in order to get the practical application in the fields of pathology.

*Dr. Talbot* (closing).—In connection with this work, six years ago, it occurred to me that a symposium each year upon the dental pulp would be a valuable contribution. About thirty years ago I realized that there were other conditions that assisted in the decay of the teeth than those that have been brought out by Professor Miller. My studies in degeneracy in this country and in Europe have taught me that in degenerates decay of the teeth takes place much more rapidly than in healthy individuals. The same thing is true in families of four or five children where all but one child is healthy. The teeth of the degenerate child decay much more rapidly than do those of the others. My work for the last thirty years has to a certain extent been along that line. My research work has taken such a great part of my time that I have not been able to prepare papers that would have pleased me to have written. With so much of my work in that direction finished, I am now taking up the study of the dental pulp, or the nervous phenomena in decay of the teeth. We have had views expressed in regard to the pulp, and have been able to find out what has been done in these directions. That is the reason we have invited gentlemen from abroad to read papers or send them to us. They are specialists along certain lines. They have advanced ideas on certain subjects, and while they do not agree with each and every one of us, yet I would just as soon have a paper from a man who has fixed ideas, whether they are correct or not. Such papers create a discussion and the happiest feeling, and assist greatly in elucidating facts. My paper to-day, as I have said, covers work of four years, and Mr. Constant has rather anticipated me in my work that I have on hand, which to my mind will bring out certain points in relation to the teeth as suggested by him and Dr. Eames. I shall present it very soon, and expect to show certain results. It demonstrates conclusively the vasomotor system of the pulp. Had I undertaken this work ten or fifteen years ago, it would have been impossible for me to have shown the pictures I have because the modern stains have developed an idea of the terminal structures of the nervous system which could not have been done years ago.

An exhibition of slides was given, demonstrating the papers of Drs. D. E. Causch, Brighton, England, on "The Development of Hard Tissue in the Pulp;" M. H. Fletcher, of Cincinnati, on "Tolerance of Tissues to Foreign Bodies, with Special Reference to the Pulp and Gums;" Martha Anderson, Moline, on "Notes on Pulp Technic;" Michael Morgenstern, Strassburg, Germany, on "Some Histologic Facts that contradict the Generally Accepted Odontoblast Theory."

Dr. Anderson's paper was read by Dr. Talbot.

(For Dr. Anderson's paper, see Vol. XXIV., page 449; for Dr. Fletcher's paper, see *ibid.*, page 596; for Dr. Morgenstern's paper, see page 161.)

Dr. A. E. Baldwin, of Chicago, Dr. M. H. Fletcher, of Cincinnati, and Dr. W. E. Walker were appointed a Nominating Committee.

Adjourned to Wednesday, May 6, at two P.M.

A paper entitled "Professional Responsibility" was read by Dr. A. E. Baldwin, of Chicago.

(For Dr. Baldwin's paper, see Vol. XXIV., page 673.)

DISCUSSION.

*Mr. T. E. Constant*, Scarborough, England.—I think that short papers on such practical every-day questions as this raised by Dr. Baldwin are extremely valuable. I agree with him more particularly in his remarks upon the necessity for the care of children's teeth. We as dentists recognize the extreme importance of the care of children's teeth, but I regret to say that in my country, and perhaps also in yours, the majority of the members of the medical profession do not systematically examine the teeth of children placed under their care and, when it is necessary, recommend them to consult a dentist. I am quite sure that if that were done many infantile troubles would be avoided, and by preserving the temporary teeth a great deal of work subsequently done by dentists in the way of regulation of permanent teeth would be rendered unnecessary.

Then, too, there is the point of the troubles dependent upon neglected conditions of the dental organs in adults. When connected with the general hospital I had many opportunities of seeing incipient gastric ulcer. In almost all of these cases the teeth were in very bad condition. In the prehemorrhagic stage of gastric ulcer, patients treated in the general hospital were relieved without any attention to the mouth, but with careful dieting, etc. It is the experience of physicians in England—I have not been able to demonstrate it—that careful repair of dental decay, removal of suppurating roots, and careful attention to the hygiene of the mouth has cured the patients without further medicinal treatment, and that so long as the aseptic condition of the oral cavity was preserved by the patient after leaving the hospital the gastritis has not recurred. It seems, perhaps, a bold thing to say, but in my opinion the majority of cases of severe gastritis in young women is due to defective oral hygiene. I therefore think the point in the paper is well taken.

*Dr. Gilmer.*—I think it very important that the attention of the members should be directed to this subject. The suggestions of the paper have been sufficiently broad to open up a wide discussion. I feel more and more that we do not get hold of children sufficiently early. As soon as the teeth have erupted we ought to see the children regularly for examinations. We frequently have children brought to us with the teeth decayed to the extent of an uncovered pulp, and with a child of nervous temperament it is no wonder that extractions are made. I therefore insist that the children shall be brought to me, not only to examine for decay, but to see whether the caretaker is giving proper attention to the dental organs and to the mouth. I believe I have in this way saved many more teeth than by filling. Prophylaxis, if intelligently carried out, will do a wonderful amount of good. We should examine not only the dental organs of the patient coming for examination, but every part of the oral cavity. If we do otherwise we are little more than beginners. If the laryngologist examined only for one diseased condition, he would hardly be considered a valuable man to the profession. Not long since, in examining the mouth of a patient, I discovered under the tongue a stone in Wharton's duct. If allowed to go on this might have been the cause of a serious condition.

*Dr. Eugene S. Talbot,* Chicago.—If there is any one time in the life of the child when oral hygiene should be at its best it is at the time of the eruption of the temporary teeth. In my studies along the line of evolution on which I have written so much it is noted that the face and the jaws and the teeth are changed from generation to generation, and that we can judge to a certain extent of the nationality of an individual by the evolution of the head and face. Therefore, I believe that in these transitory structures the conditions in the mouth, decay of teeth, etc., are very apt to occur. It is at the time of the eruption of the temporary teeth that we have what I have termed the second period of stress. These periods of stress are very much on the same order as the systemic changes every seven years the old people speak of. At this time the dentist has an idea that all diseases of the human body revolve around the teeth, and to a certain extent this is true; but he forgets that the mucous membrane throughout the body is undergoing a great change at this time. Up to this time the child has been taking liquid food, and now the system is preparing itself for harder food, and the cells of the mucous membrane are undergoing a great change. Therefore, as the essayist has stated, the greatest care of the teeth should be taken at this time. I am not in harmony with Weissman regarding the inheritance of acquired defects. I do believe in the inheritance of acquired defects, and have proved the theory. In Mississippi, and I suppose in other parts of the South, and in England, the habit is to extract the first permanent molar, especially if it is decayed. In the South it is customary to extract it on general principles. The reason is that this tooth comes in irregularly, and by its extraction the balance are allowed to come in in normal condition. This particular point is important because of the transitory nature of the jaws; this has a tendency to produce arrest of development. It is therefore a cause of irregularities of the teeth seen in England which is further intensified from generation to generation.

*Dr. Curtis.*—Mr. Constant's remarks are well taken. I believe that the greater part of the work done by our throat specialists is necessitated by the pathological condition of the teeth. The children should be brought to the dentist for examination yearly, and he should direct to whom they should be sent for proper treatment of the affections caused by these pathological conditions of the mouth. I remember quite early in my practice a throat specialist meeting me on the street and saying, "I shall soon have to send you a percentage of my income." I asked upon what ground he made the statement, and he replied, "You send me more patients in nose and throat work than all the other dentists in the city." That stimulated me to send more, to be more careful in my examinations of the nose and throat of my patients. I did not, however, accept a percentage.

I think the extraction of the sixth-year molar is a lazy man's way of avoiding work. I believe it should never be done except where the child's tooth is abscessed, and in many cases not then. There are, however, conditions, as I said yesterday, in which you have to treat the case individually and according to the conditions of the mouth and of the patient.

*Dr. M. L. Rhein.*—At such meetings as this, the necessity for men who assume the position of caring for the teeth to realize their responsibility cannot be too strongly urged. It is my invariable rule to pay absolutely no attention to the suggestions or directions of people coming to me until I have made a thorough examination. I then inform them of what I deem advisable to be done, and if it does not meet their wishes our relations end at that point. I believe that is the only truly professional line of conduct. This, I think, differentiates between what we are pleased to call stomatologists and dentists.

*Dr. Hans Pilcher,* Vienna.—I do not think I have anything to say in this matter. I am pleased with the suggestions of Dr. Baldwin, and I shall bear them in mind in my practice.

*Dr. W. E. Walker,* New Orleans.—It occurred to me (if I may discuss the discussion as well as the paper, for I can only commend the paper) that we do not always "see ourselves as others see us." I did not know that it was the practice of England and the South to sacrifice the first permanent molar. That this extraction does occur here I am aware, but I judge from articles written on it that this is practised elsewhere. Here I know it is too common.

In regard to examining the whole mouth instead of looking simply at the tooth to which attention is directed by the patient, that this practice is not sufficiently common among dentists has been impressed upon me by the surprise of the patient manifested when I have proceeded to examine the whole oral cavity rather than simply the tooth complained of.

*Dr. M. L. Rhein.*—I thoroughly agree with Dr. Walker that there is as much extraction of the first permanent molar in the East and West as in the South. I do not believe it is confined to one section of the country, and I am pleased to put myself on record as taking the position, the exception proving the rule, that there is no warrant for the removal of the first permanent molar.

*Dr. George F. Eames,* Boston.—The subject of the essayist's paper is one of the greatest importance. I can conceive of no other that can come before this body being of so great importance as this. It has to deal with three or four items always to be considered in our professional life: (1) The relation of the stomatologist to other specialists in general medicine, or the dentist to the doctor. That is a great subject in itself, and should be ever kept in mind. (2) Another is the relation of the stomatologist to his patients. (3) The relation of the stomatologist to other dentists. (4) The relation of the stomatologist to himself. This subject should be brought to the attention of the general practitioner and to other specialists, that patients may be advised in regard to oral conditions. It should be brought to the attention of the laymen. The subject deals with the relation of the local conditions in the mouth to the general health, and this I consider of the greatest importance, far and away above other considerations.

*Dr. Baldwin* (closing).—Of course, in a paper of this kind one can do nothing more than touch upon the different subjects. I would especially emphasize what Dr. Gilmer has said in not having patients sufficiently early.

As Dr. Eames has said, there are relations to be recognized. Our object should be to unify all the departments of the healing art in one great whole.

I thank you for the discussion, and realize more than any one the shortcomings of the paper.

A paper by Dr. O. N. Heise, of Cincinnati, entitled "Empyema of the Antrum," was then read.

(For Dr. Heise's paper, see Vol. XXIV., page 608.)

DISCUSSION.

*Dr. Gilmer,* Chicago.—Mr. Chairman and gentlemen of the Section, I had the pleasure of looking over this paper previous to its reading, and I feel that it is a valuable paper, although the field which it covers is a narrow one. I feel that Dr. Heise has done well in differentiating between a pus sac in the end of the root and true empyema. I do not know whether I could agree with him in the nomenclature, but I think he has done well in the differentiation. We are often defective in diagnosis of the various diseases to which the anatomy is subject. We take, for instance, the condition spoken of here. He calls it cystic empyema; the other, true empyema. It may be that it will be pretty difficult to separate these two, although there is a vast difference. Both of them practically are encysted. We have a root of a tooth which approximates closely to the floor of the antrum, and we have a sac on the end of the root filled with pus. Some years ago this was spoken of as pyogenic membrane. Of course, there is no such thing as pyogenic membrane. There are conditions in which the bone is absorbed and the sac continues to grow, completely filling the cavity. We will have difficulty, therefore, in making a diagnosis. We will say that the patient has all the symptoms perhaps. He has the thinning of the buccal and nasal walls, but there is no appearance of pus. It may be said it is not an encysted maxillary sinus, but to all intents and purposes it is an empyema of the maxillary sinus. An empyema really means a cavity that is completely filled with pus, having no opening. Therefore, the so-called empyema of the maxillary sinus is not an empyema at all. But here we do have an empyema of the maxillary sinus because it is a sac that is filled with pus.

It is difficult to differentiate between the various diseased conditions in the antrum. Regarding the causes of the diseases of the antrum it seems to me that usually they come from only two sources. We may have syphilitic disease of the antrum. This I have seldom seen. The two sources are from the nose, the inflammation extending by the continuity of tissue into the maxillary sinus, and from the roots of the teeth. The root of the tooth is often given as the cause of the trouble when it is not primarily so. We have as a result of a catarrhal condition of the lining of the membrane an inflammation which may cause the death of the live and healthy tooth. You will find the floor of the antrum thinned out, representing the roots of the molar or bicuspid. If you will remove one of these little hillocks (indicating), you will find the merest swell. The mucous membrane overlying becomes inflamed; it may involve the pulp, and the tooth may die. The condition may not be recognized. We have a pyogenic condition at the end of the root which keeps up a slow inflammation and continues the disease of the antrum. Therefore, this trouble is secondary rather than primary. I believe we have a great deal more of the disease of the antrum since the advent of influenza in this country. We have an irritation of the lining of the antrum causing a stenosis, which in turn causes an excess of fluid, and by infection of that fluid we have an empyema. I find another condition resulting from a pulpless tooth and an antrum not fully antiseptic. We may have a small pus cavity walled off. The inflammation of the lining membrane comes from the continuity of the tissue from the nose, and results in an empyema of the maxillary sinus, which we would not have had we not the inflamed tissue. My experience teaches me that in the case of a root of a tooth in which the pulp is dead extending into the antrum, nothing will cure the condition but the removal of the tooth. If an opening is required I would not make it from the antrum, but from the nose. If the case demands an operation upon the antrum, a broad surgical treatment should be carried out. If I should make the opening just above the membrane of the gum, I would expect to find in people between forty and fifty the bone very thin. I would make the opening sufficiently large to insert my little finger, receiving an impression of the condition. Another reason for opening here (indicating) is that the cheek falls in. An opening here allows also the insertion of an electric lamp into the maxillary sinus and permits a thorough washing out.

It may be that Keil erred in attributing so many cases of the teeth as a cause of disease of the antrum because he did not recognize that here was a secondary cause in the pulp of the tooth having been destroyed. He says that they are the largest factor in etiology. That may be true; not, however, primarily, but secondarily.

It seems to me that we ought almost never use arsenic for the destruction of pulps. The application of cocaine is very much better. There are sometimes accessory or additional openings in the roots of teeth larger than the apical openings, and through these the arsenic may do much harm. I cannot conceive why chloride of zinc or carbolic acid should be used in such cases.

*Dr. M. H. Fletcher,* Cincinnati.—This subject is one which has interested me very greatly and one upon which I have had a great deal of worry. I have had many cases in which I have blamed myself for not being able to fully understand. I find, however, that my experience is the duplicate of many others. I would like to speak of some of the features of the anatomy spoken of by Dr. Gilmer. In 1891 or 1892 I took occasion to go through the Army Medical Museum to study the anatomy of the antrum, and it turned out, in the examination of fourteen or fifteen hundred antra, that the descriptions by anatomists known to me at that time were erroneous. In other words the anatomy as pictured by Dr. Gilmer and pictured by anatomists was found in twelve skulls out of the fifteen hundred. On the other hand, when we came to the connection of the floor of the tooth with the floor of the antrum, I found in a large percentage of cases that there was no bony covering to many roots of the teeth. Instead of being thin and parchment-like, you will have an opening here, the apex of the tooth coming against the soft tissues of the antrum. That being the case you can easily see the condition spoken of here. I have seen several cases of such perforation and an abscess discharging into the antrum in which healing took place as readily as if there had been an opening by the alveolar process. We all know that on the buccal surfaces many of the roots are perfectly bare of bone, being simply covered by a mucous membrane, and, as I have stated, many cases are so in the antrum. An abscessed tooth coming in contact with the antrum in that way would discharge in the antrum and the pus go in the way of least resistance. Fortunately, the majority discharge into the mouth. It is my experience that pulpless teeth producing antral trouble can be made to heal as readily as though they discharged into the mouth. On the other hand, my recent experience in treating these cases has confirmed me in the belief that diseases of the alveolar process and calcareous deposits are more largely to blame for antral trouble than pulp lesions or the loss of the pulp. It is natural from my reasoning as to pathology that a diseased bone about the tooth can much more readily cause diseased antrum from continuity of tissue than a diseased pulp producing an abscess. If you have necrosed bone about the tooth you must have inflammation extending in every direction, and the bone extends upward, towards the antrum. The extension of dead bone and inflammation is apt to be very great if it has entered to any considerable distance into the alveolar process. It never has been my experience to see a bicuspid that came very near to the floor of the antrum as compared with the molars. I have seen these openings referred to by Dr. Gilmer.

I have talked with Dr. Heise about antral trouble from the live tooth. I fail to see any condition about the tooth with a vital pulp that would cause antral trouble. I can conceive of an exostosis producing some inflammation, but if the pulp is alive I do not see any reason why it should cause antral trouble or produce pus in the antrum.

The use of arsenic is a pet scheme of mine. I have used it about ever since I have been practising, and it is my habit, if I feel that in any way I have been inefficient in opening the root-canals, to insert some arsenic and leave it, expecting it to produce asepsis. I have done this not only a few times, but a great many. I do not, however, use arsenic to any degree in destroying pulps.

*Dr. Belden.*—In view of the fact that you use arsenic at all, what is your objection to using it in destroying pulps?

*Dr. Fletcher.*—If we use enough of it to destroy the pulp in a reasonable time, it is sufficient to cause sloughing afterwards. As to the use of zinc and other medicaments that destroy bone, I have seen carbolic acid destroy quite a large area of the alveolar process. On the other hand I never have seen arsenic do it. Arsenic, however, if applied to live tissues, will probably destroy a small zone. It never has in my experience produced a necrosis. It may produce destruction, but that may be compared to traumatic injury. The tissues are dead. They do not continue to die and necrosis ensue. Also, it has been my experience that the tissues will remain sore for months, may be for years, if the application is short of destruction of tissue. There is, however, no septic condition, no necrosis, no pus. The arsenic is so aseptic that it stops that process.

*Dr. Gilmer.*—Have you not seen the destruction of bone between the teeth from the use of the arsenic?

*Dr. Fletcher.*—I never have.

*Mr. Constant.*—I have on three occasions.

*Dr. Gilmer.*—I have seen it many times.

*Dr. Fletcher.*—The author has distinguished between the cystic condition and that of empyema. I think I have to stretch my imagination quite a little bit to think a tooth would produce that trouble. I would think such trouble more like adenitis than a pus cavity.

*Mr. Constant,* Scarborough, England.—I would speak of two points; first, with regard to the etiology. As a student I was much impressed with the importance of the tooth in the causation of empyema of the antrum. It is my experience that the longer one is in practice the less one feels inclined to suspect the tooth as the cause of empyema of the antrum. In my own experience it has been very exceptional to be able to find a case in which disease of the antrum could be definitely traced to the tooth.

In regard to what Dr. Gilmer says of the difficulty in distinguishing between secondary trouble in the tooth after the empyema,—that is, in determining cause and effect,—I am quite sure that in many cases of so-called empyema, put down as due to abscess connected with the tooth, is putting the cart before the horse. I can recall two cases in which empyema of the antrum was supposed to be due to trouble in the first molar. When the first molar was removed, it was found possible to evacuate the pus, and that was taken as proof that the tooth caused the trouble. I could not convince myself that the tooth was the cause of the trouble. I think the fact that the apices actually went into the antrum made it easy to get the discharge in that way. On that point I quite agree with Dr. Fletcher that it is far more common to find the apices of teeth bare within the antrum than to find them covered. A great number which I have examined in England show that.

The other point is the distinction drawn between so-called cystic conditions within the antrum and pus in the antral cavity. In one case, in connection with the second bicuspid, such a condition was present. When the second bicuspid roots were removed there was a very copious discharge of aqua-sanious fluid which kept discharging. The antrum must have been quite filled with it. The sac gradually contracted and healed in less than three weeks, and there was no further trouble.

*Dr. M. L. Rhein.*—I feel convinced, from a careful study of this subject, that too often a mistake in etiology is made in these cases, and that the cases in which the tooth is the primary etiological factor are very very rare. The tooth becomes involved through pathological conditions between the end of the root and the antrum, and is too often given the blame. I think it is wise to emphasize this point.

*Dr. Fletcher.*—I think Dr. Talbot and I found that the molar tooth could produce this trouble in about five per cent.

*Dr. A. E. Baldwin.*—I am a firm believer that arsenic can seldom if ever affect the tissues beyond the pulp of the tooth. Dr. Gilmer has referred to the septum being lost by the careless application of the arsenic in the particles getting down between the teeth.

*Dr. Gilmer.*—I spoke of the particles on the sides of the root which I have found and been able to pick off.

*Dr. Baldwin.*—Years ago I suspected the same thing, and I made several examinations in this way: In a central incision I would make application to the pulp of the tooth, and after a sufficient time I took out the pulp. I then took several sections of the pulp, but could find no evidence of the arsenic in the several parts. I do not think, therefore, that the pulp could convey the arsenic to the adjacent tissues. I think a great deal of the injury is done by the careless application of the arsenic and the ease with which it gets outside of the tooth, causing the destruction of bone and soft structures.

*Dr. Curtis.*—The anatomical features have been beautifully brought out. I would just like to say that I think there are very few diseases of the antrum of a primary nature. I think the large majority of diseases of the antrum are due to affections of the teeth. Some reasons for slow results in treatment are on account of a tuberculous condition. I think the paper is particularly valuable in the matter of differential diagnosis. The cystic conditions usually found in the antrum I believe are largely due to alveolar abscess, to septic conditions of the pulp, or of the canal. I have seen some cystic tumors where there was very extended absorption of the bone, and where the periosteum of the antrum was so crowded to the opening at the nares that there was actually no antrum left. This was one of the most serious cases of antrum affection that I have seen.

(To be continued.)

