# Correction of Stenographer’s Reports

**Published:** 1904-04

**Authors:** Thos. L. Gilmer


### CORRECTION OF STENOGRAPHER'S REPORTS.

To the Editor:

Sir,—I have just seen in the International Dental Journal the discussion on Dr. Heise's paper, "Empyema of the Antrum," read at the meeting of the American Medical Association, and am humiliated with the report of my part of the discussion. Parts of the report are so bad as to be uninteligible. In some instances words, parts of sentences, and whole sentences are omitted; which spoils the sense; in others the stenographer's report, which was very bad, has been edited so as to make me say things I did not say. A few of the errors I note.

Line 5, page 212, should read pus sack *on* the end of root, not pus sack *in* the root.

Line 9, page 212, should read to which the *antrum* is subject, not to which the *anatomy* is subject.

Line 33, page 212, I am made to say that I have *seldom* seen syphilis of the antrum. What I said was that I had *never* seen syphilis of the antrum.

Lines 12, 13, 14, page 213, as they stand are without meaning.

Lines 20, 21, page 213, I am made to say, if an opening is required, I would not make it from the *antrum,* but from the *nose.* What I said was, if only drainage was needed I would make the opening from the *nose,* but if curettement was necessary I should make an opening from the *buccal wall* of the cavity.

Line 23, 24, page 213, should read at the *duplication* of the mucous membrane and the gum, not just *above* the membrane of the gum.

Lines 27, 28, page 213, Another reason for opening here is that the *cheek falls* in, should read, because if the opening is made through the buccal wall the cheek *falls over the opening* and in a large measure prevents food from getting into the cavity, etc., etc.

In justice to myself and Dr. Heise, I request that this communication appear in the April number of the JOURNAL, and that with it you publish my part of the discussion as it apppeared in the January number of the *Journal of the American Medical Association,* page 25, a copy of which is enclosed. I regret that a copy of the stenographers report from which your report was made had not been, as was that of the *Journal of the American Medical Association,* sent me for correction. In this correcting I made no changes except to correct the stenographers errors and condense the matter in a few instances.

By kindly complying with the above request you will greatly oblige,

THOS. L. GILMER.

[Our Correspondent is in error in supposing changes were made by the editor of this journal in the discussion referred to in the foregoing communication. The report was received at this office from the official stenographer of the Section on Stomatology, American Medical Association, and supposed to be practically correct, the editor having no means of determining this question except through the very uncertain method of sending proofs of the discussion to each one taking part. This the INTERNATIONAL cannot undertake to do, unless it is specially requested.

A fair examination of the errors noted, while annoying, are not so far out of the way but that any intelligent reader could make the corrections. Our correspondent does not allow for this. Every speaker is subject to these minor errors in reports.

The stenographer receives the blame when it more often is the fault of the speaker. Some get accustomed to these errors, others do not.—Ed.]

